# The dynamic threat from landslides following large continental earthquakes

**DOI:** 10.1371/journal.pone.0308444

**Published:** 2024-08-21

**Authors:** Katherine Arrell, Nick J. Rosser, Mark E. Kincey, Tom R. Robinson, Pascal Horton, Alex L. Densmore, Katie J. Oven, Ram Shrestha, Dammar Singh Pujara

**Affiliations:** 1 Institute of Hazard, Risk, and Resilience and Department of Geography, Durham University, Durham, United Kingdom; 2 Department of Geography and Environmental Sciences, Northumbria University, Newcastle-Upon-Tyne, United Kingdom; 3 School of Geography, Politics and Sociology, Newcastle University, Newcastle upon Tyne, United Kingdom; 4 School of Earth and Environment, University of Canterbury, Christchurch, New Zealand; 5 Terranum SARL, Bussigny, Switzerland; 6 National Society for Earthquake Technology, Kathmandu, Nepal; Guizhou University, CHINA

## Abstract

Earthquake-triggered landslides show three important characteristics: they are often responsible for a considerable proportion of the damage sustained during mountain region earthquakes, they are non-randomly distributed across space, and they continue to evolve in the years after the earthquake. Despite this, planning for future earthquakes rarely takes into consideration either landslides or their evolution with time. Here we couple a unique timeseries of mapped landslides between 2014–2020 across the area of Nepal impacted by the 2015 M_w_ 7.8 Gorkha earthquake and a numerical landslide runout model overlain with building locations to examine how the distributions of both evolving landslide hazard and exposure intersect to generate a dynamic threat to buildings. The threat from landslide runout is shown to change in predictable ways after the earthquake, becoming more pronounced at mid- and lower-hillslope positions and remaining in the landscape for multiple years. Using the positions of our mapped landslides as a starting point, we can identify *a priori* the locations of 78% of buildings that were subsequently impacted by landslide debris. We show that landslide exposure and hazard vary from negligible to high, in relative terms, over lateral distances of as little as 10s of m. Our findings hold important implications for guiding reconstruction and for taking steps to reduce the risks from future earthquakes.

## Introduction

Strong ground shaking from moderate- to high-magnitude continental earthquakes in mountainous regions can initiate a chain of hazardous Earth surface processes that can far outlast the initial event itself [1–3]. One consequence is that communities and authorities engaged in and responsible for reconstruction must deal with this legacy of latent risk for months or even years [4].

However, a detailed analysis of how the legacy of earthquakes in mountain regions shapes this geography of risk, and specifically how post-earthquake geohazards impact people, is currently lacking. This results from limited time-series data describing post-earthquake risk that span the dynamics of both landslides and people’s locations in the landscape. Importantly, it is known that hazard and exposure can vary considerably over short distances [5], and so risk can be highly heterogenous. These limitations have rendered trends in risk difficult to identify and hence limit more holistic planning for future earthquakes [6–10].

Nepal is an example of a mountainous country that is affected by both large earthquakes and monsoon rainfall. Aseismic landslide activity in Nepal is predominantly triggered by the Asian Summer Monsoon during June to September, when intense and persistent precipitation triggers new landslides, further destabilizes existing landslides, and readily remobilizes poorly consolidated sediments [4, 11]. Aseismic landsliding in Nepal has claimed at least 1,400 lives since 2011 [12, 13] and results in long-term disruption to settlements and infrastructure, with annual average damages of ~US$1 billion [14]. Superimposed upon this are the acute impacts of earthquakes, including the M_w_ 7.8 Gorkha event in 2015, which triggered >25,000 landslides [15], resulted in >9,000 deaths [16], and impacted >8 million people, accruing damages of more than US$7 billion [17]. Continuing post-earthquake landslide activity has been shown to exceed pre-2015 levels [1, 18, 19] overprinting the impacts of a rapidly expanding rural road network [11, 14, 20–22], rapid development and reconstruction [23–25], and interannual variability in monsoon precipitation [11, 26].

As the future impacts of landslides associated with monsoons and earthquakes are likely to grow [14, 18, 27–34], a better understanding of their associated risks is essential [28]. Mapping studies of landsliding immediately following the Gorkha earthquake provided an instant barometer of landslide hazard (e.g., [15]) and enabled the constituents of post-seismic landslide hazard to be disaggregated [4]. However, while there has been some consideration of persistent risk to, for example, major highways (e.g., [19, 35]) and dams (e.g., [36]) there has been little attention to the specific risk posed by post-earthquake landsliding, or by reworking of material released during the earthquake, to life-critical assets such as housing. To our knowledge, there has been no systematic assessment across the entire earthquake-affected area of the changes in building-level risk due to both new post-earthquake landslides and to remobilization and runout of landslide debris. Critically, co- and post-seismic landslides occur in distinct, predictable locations across the landscape [1, 3, 5, 37, 38]. Similarly, settlement patterns reflect complex but often distinctive choices related to land tenure and ownership, culture, and livelihoods, and so often display typical configurations within and across the mountain landscape [23]. Assessing how these non-random distributions of hazard and exposure intersect after an earthquake is vital in mapping risk, particularly given that both can be highly dynamic in time and variable over small distances [28, 39].

**Fig 1:**
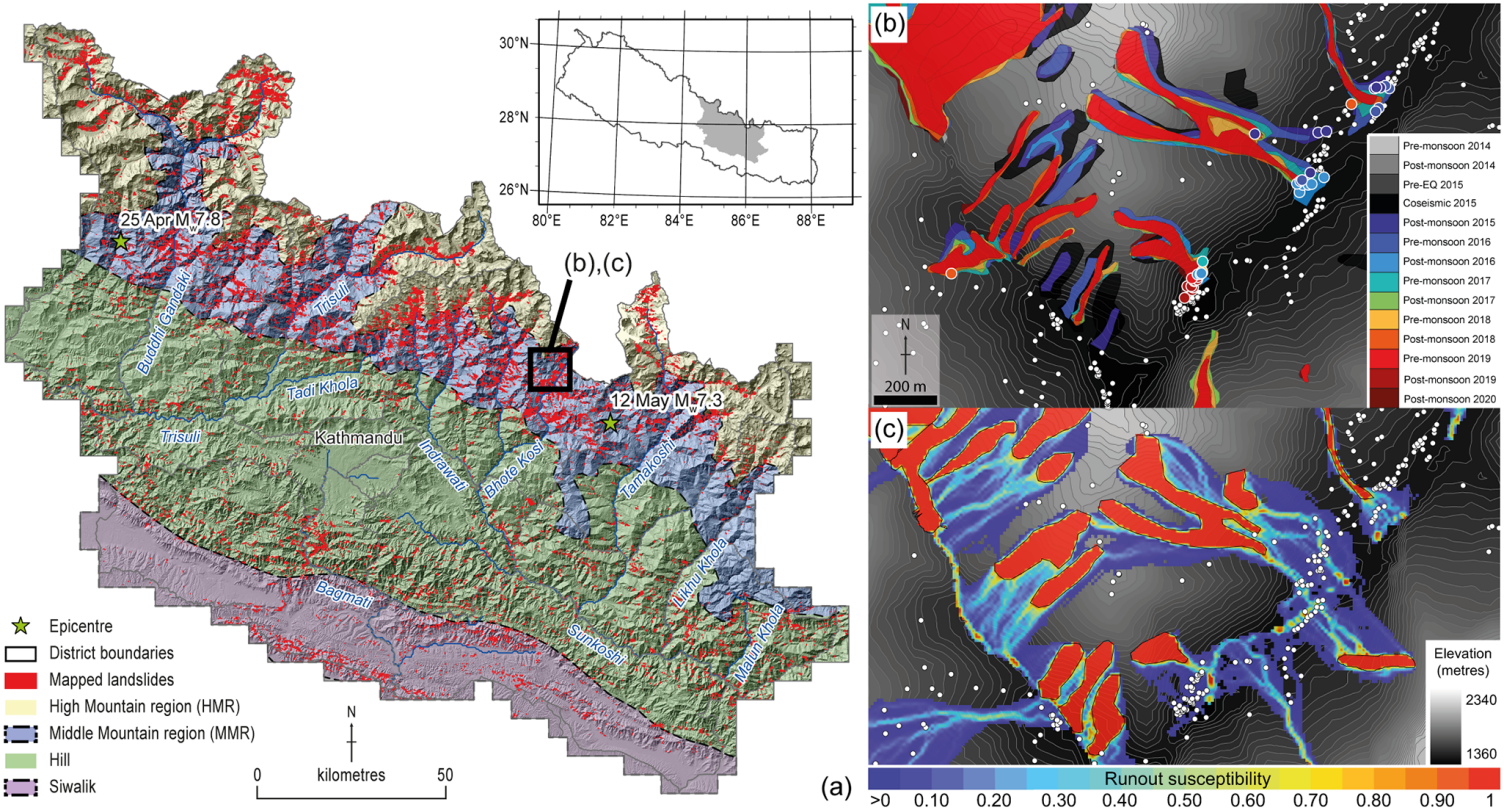
Study area location map and subset maps showing landslide inventory, runout susceptibility and building data. **a)** Location of the study area in central Nepal (area = 24,402 km^2^), showing district (black) and main physiographic boundaries based on [43]. Stars show the epicentres of the 2015 M_w_ 7.8 Gorkha earthquake mainshock that occurred on 25 April as well as the M_w_ 7.3 aftershock on 12 May. Red polygons show the mapped landslides across all epochs in 2014–2020 from [1]. **b)** Mapped landslides from the 14 inventories from 2014 to 2020 plotted with building locations, showing the elongation and growth of some landslides in the five years following the 2015 Gorkha earthquake. Coloured circles show buildings where runout was predicted and were later impacted by landslides; the colours indicate the epoch in which the impact occurred. The inset area is shown in panel (a). **c)** Flow-R landslide runout susceptibility values from coseismic landslides, ranging from 0–1, overlain with building locations from our building catalogue dataset (white circles). Red polygons show mapped coseismic landslides; runout susceptibility in these locations is equal to 1 because they are already impacted by landsliding. Grey background contour areas have runout susceptibility of zero, because they do not lie downslope of mapped landslide source areas. Note that runout susceptibility was calculated separately for each of the 14 landslide inventories; only runout susceptibility from the coseismic inventory is shown here for clarity. The topography shown is derived from the Shuttle Radar Topographic Mission (SRTM) 1 arc-second ~ 30 m DEM (Credit: USGS EROS Archive), which has a lower spatial resolution than the 10 m AW3D DEM used to calculate landslide runout susceptibility.

Here we examine the threat posed to settlements by landsliding in the five years following the 2015 Gorkha earthquake. Utilising landslide time-series inventory data [1] and a distributed physics-based run-out model with a precautionary parameterisation [40, 41], we predict how the runout of landslides triggered both in and after the earthquake intersect a catalogue of 1.1 million buildings. Across the earthquake-affected area (Fig 1a), we identify emergent spatial and temporal patterns in the threat from landslides as a direct function of the evolution of landslide hazard. Critically therefore, we consider both buildings where impacts occurred, where mapped landslides (both those that evolve, and those that newly occur) have impacted buildings and threat was realized, and buildings under threat where modelled runout from existing landslides intersects buildings, identifying those potentially under threat in the future. The uncertainty of threat posed by runout from existing landslides is considerably lower than that from potential new landslides, so separating these provides insight into the changing certainty with which under-threat buildings can be identified. We use these data to assess: (1) how the spatial evolution of landslide hazard affects the generation of threat from landsliding across the landscape after an earthquake at regional and hillslope scales; (2) the time scale over which landslide hazard and threat evolve; (3) the degree to which different forms of post-earthquake landslide hazard and threat can be predicted; and (4) the spatial scales over which exposure to threat varies and the implications of these for post-earthquake risk reduction.

Within this study we use extracts of the definitions adopted by the United Nations Office for Disaster Risk Reduction (UNDRR) [42]. *Hazard* here is defined as a process that may cause property damage and within this study is represented as (i) the areal footprints of mapped landslides and (ii) areas within the landscape found to be susceptible to future landslide runout. *Runout susceptibility* is defined and calculated as the quantitative likelihood of an area being impacted by the future runout of debris from a mapped landslide. *Exposure* is defined as the situation of building structures located in hazard-prone areas and within this study is represented as point-based building locations. Within this study we use the term *threat*, rather than *risk*, to describe the intersection of these two datasets, where *hazard* and *exposure* are spatially coincident. No temporal constraints on runout susceptibility are calculated within this study.

## Study area and methods

### Study area

The Himalaya in Nepal have developed through ongoing collision between the Indian and Eurasian plates since the early Cenozoic (e.g., [44–47]). This collision is accommodated by thrusting along the Main Himalayan Detachment and a series of splay fault systems that branch upwards from the detachment. The topography of Nepal reflects this ongoing collision, with a northward increase in mean and peak elevations from the Siwalik foothills in the south along the active Main Frontal Thrust to the Hill region in central Nepal and then the Middle Mountains and High Mountains regions in the north (Fig 1a).

The Gorkha earthquake occurred on 25 April 2015, with a moment magnitude of M_w_ 7.8 and an epicentre 80 km west–northwest of Kathmandu, beneath the front of the High Himalaya (Fig 1a). The earthquake focal mechanism was dominated by thrusting on a sub-horizontal strand of the Main Himalayan Detachment fault dipping ca. 10° northwards with a hypocentral depth of 15 km [48]. The largest aftershock (M_w_ 7.3) occurred near the eastern end of the mainshock rupture plane [49] (Fig 1a). The area of Nepal that experienced significant damage was declared by the Government of Nepal as the ‘14 earthquake-affected districts’, covering an area of approximately 28,300 km^2^ of Central and Western Nepal and extending from the Siwaliks in the south to the High Himalaya in the north (Fig 1a). Whilst estimates of landslide number vary based on mapping technique [1], around 25,000 were triggered by the initial shaking [15], with higher densities focused in an east-west swath to the north of Kathmandu. A more limited number of studies have identified patterns in how these landslides have since evolved, and show a clear role of topography and landscape position but a lesser control of bedrock geology [19, 40].

The landslides triggered included a range of types, including rockfalls, debris slides, rock avalanches, large rock slope failures and debris avalanches, and deeper-seated rotational slides. Since the earthquake, and notably after each successive monsoon, considerable volumes of debris have been mobilised and reworked, with debris slides and debris flows running out into the drainage network from coseismic landslides, new landsliding in areas that may have been damaged by the earthquake, and ongoing movements of long-established landslides in the landscape, as has been observed both within the study area [19, 50, 51] and after other mountain region earthquakes [7, 52, 53]. Mapping from satellite imagery and field investigations have monitored this evolution to identify systematic spatial and temporal patterns in how these landslides, and their behaviour, evolve [54]. Other studies have sought to explain the variability in year-on-year landsliding rates as a function of monsoon strength [37] and development impacts such as rural road construction [55]. It is notable that the complexity of how these landslides change in space and time, and limited data on potential causative factors (e.g., rainfall at sufficiently high spatial and temporal resolutions), make attributing clear controls on the pattern of landsliding challenging.

### Methods

#### Landslide inventory mapping

The methodology used to generate the landslide inventories was described in detail by [1]. Landslide mapping was based on visual interpretation of true colour (RGB) and false colour (NIR) composite medium-resolution satellite imagery between 2014 to 2020, to manually delineate landslide extents. Landsat 8 imagery with a spatial resolution of 30 m was used for 2014 to 2015 with multispectral bands pan-sharpened to 15 m using the panchromatic band 8. Sentinel-2 imagery with a spatial resolution of 10 m was used for 2016 onwards. Thus, the effective minimum landslide area in the inventories is on the order of several hundred m^2^, with a pronounced rollover in the area-frequency distribution at an area of c. 2000 m^2^ [1]. In total, 14 discrete epochs were mapped, comprising annual pre- and post-monsoon inventories for 2014–2019, an additional 2015 coseismic inventory, and a single post-monsoon 2020 inventory. In each epoch, all visible landslides were mapped, regardless of whether they were present within an earlier inventory (Fig 1b) [1], creating a multi-temporal inventory of 199,350 mapped landslides.

#### Landslide runout modelling

Flow-R models the potential runout of material from defined landslide source areas, which here are the mapped landslides from each of our multi-temporal inventories; the runout path and extent are controlled by a spreading algorithm and friction parameters [41]. The model was parameterised in a precautionary manner to consider a worst case, deemed appropriate given: (i) a lack of widespread empirical data on post-earthquake runout for *a priori* calibration, and (ii) the need for a risk-averse strategy to inform post-earthquake risk assessment [4, 40]. Potential runout from each of the 14 landslide inventories was sequentially modelled across a 10 m digital elevation model (DEM) subsampled from the 5 m JAXA AW3D dataset. For each 10 x 10 m cell, the model generates a runout susceptibility value in the range (0,1), with larger values indicating locations that are more likely to be within a runout pathway–in other words, locations that are more likely to be affected by landslide debris that is subsequently remobilised and transported downslope (Fig 1c). Locations that fall under a mapped landslide have runout susceptibility equal to 1, because they are already impacted by that landslide (Fig 1c). Because Flow-R does not explicitly track mass downslope, it is important to recognise that runout susceptibility is not time-dependent, and there is therefore no information on the downslope velocity of material or when debris will reach a given point in the landscape [e.g., 38]. Flow-R software version 2.0 was used for this study, with full details of the Flow-R methodology provided within [40].

#### Landslide threat mapping

**Fig 2:**
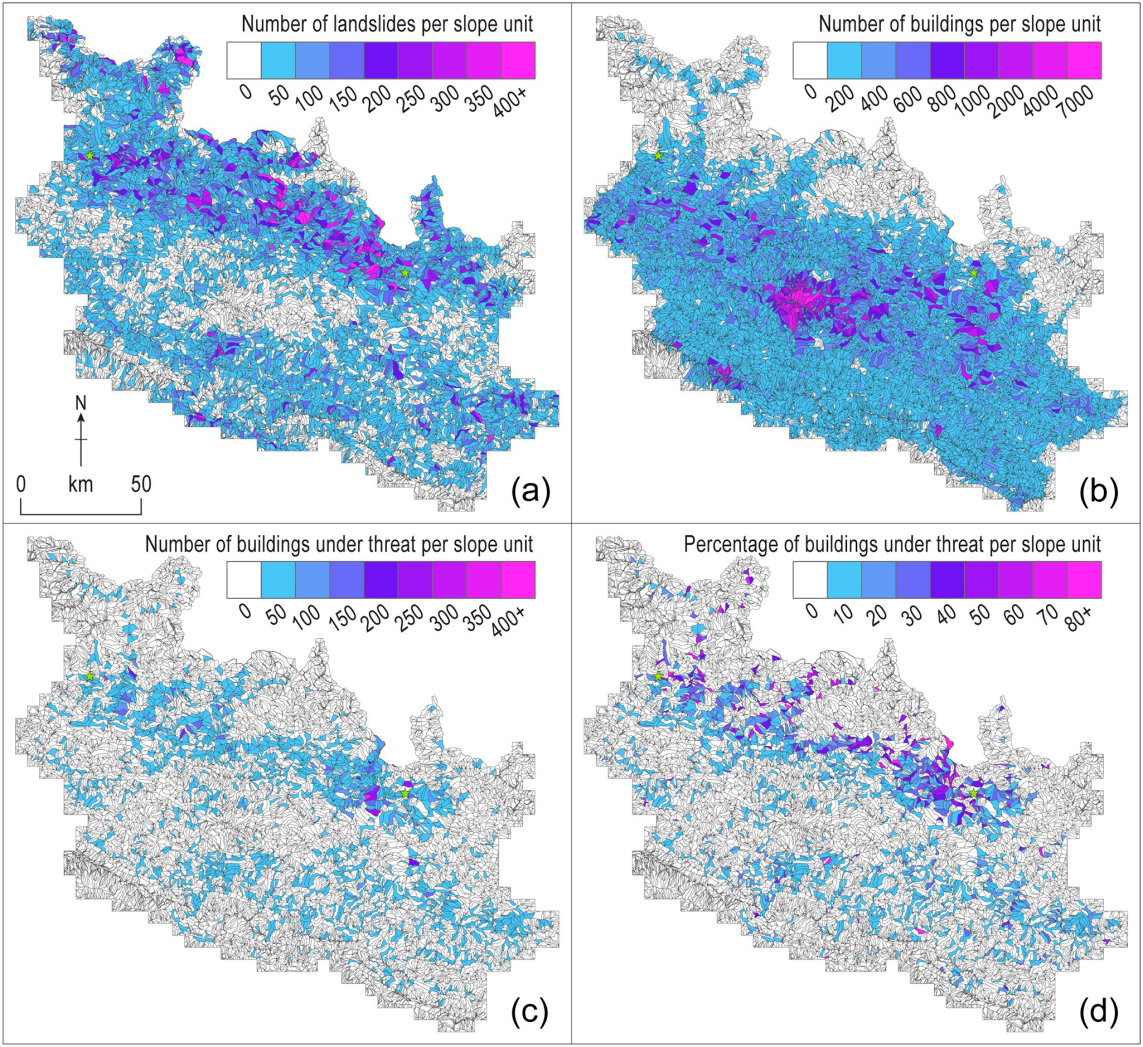
Geographical trends in hazard and the consequent threat to buildings within the study area. **a)** Number of mapped landslides across all 14 mapping epochs displayed by slope unit. **b)** Number of buildings per slope unit. **c)** Number of buildings identified to be under threat in any epoch per slope unit. **d)** The percentage of buildings per slope unit identified to be under threat in any epoch.

A static point-based building catalogue dataset from the Department of Survey (Government of Nepal) was supplemented with the centroids of polygon building data from Open Street Map (OSM) data (Fig 2). These static data represent the most accurate spatial information on the location of buildings at a national scale. Because we have no corresponding data on building type, construction, or fragility, we assumed each building to be equally vulnerable to damage. Thus, threat was assumed to be simply and directly proportional to landslide runout susceptibility, as modelled by Flow-R [56]. No information is available on any buildings that were subsequently damaged, destroyed or rebuilt.

The modelled runout susceptibility value was extracted for each building location for every epoch; this value was considered as our building threat score. Multiple buildings within the same 10 x 10 m DEM cell were assigned the same runout susceptibility, and thus the same threat score. Conversely, where a building lay in the path of runout from multiple landslides, the maximum runout susceptibility recorded at that location per epoch was used. Threat scores were classified into 4 categories: 0 = no threat, > 0 to < 0.1 = low threat, ≥0.1 to < 0.5 = moderate threat and ≥ 0.5 = high threat.

To provide a geomorphologically meaningful discretization of the landscape over which to aggregate the changing threat from landslides, we defined slope units using the methodology outlined by [57]. Slope units partition a landscape based on hydrological and geomorphological boundaries, such as ridge lines and breaks in slope, and generally provide a more appropriate unit of summary analysis across large spatial extents than regular fishnet grids. Using the maximum and minimum elevations within each slope unit, a normalized elevation (denoted as *Zˊ*_*SU*_) was calculated for each DEM cell with a value in the range [0,1] to enable more meaningful comparison of landscape-scale building position irrespective of the scale of the topography.

The positions (defined by *Zˊ*_*SU*_) of all buildings, under-threat buildings and per epoch under-threat buildings were compared using kernel density estimates to identify any differences in their distributions with *Z’*_*SU*_. Following the methods outlined by [1], kernel density estimates were calculated in the same way for all populations of buildings, to ensure that comparisons could be made. Each density estimate was calculated at intervals of 0.001 between *Zˊ*_*SU*_ values of 0 and 1, with a moving window half-width, *h*, of 0.03. Differencing density estimates (for example, between under-threat buildings and all buildings) allows us to identify slope unit positions with relatively more (positive values) or less (negative values) under-threat buildings than would be expected from the overall building distribution.

Validation of the areas of modelled debris runout after the 2015 earthquake was performed and reported in [40], but here we extended this validation by also analysing the threat history of buildings that were impacted by that runout. Modelled building threat was validated using observations of buildings that fell within the footprint of mapped landslides in a later epoch. Validation was calculated per epoch and did not consider direct impacts on buildings within newly-triggered landslides, which were not analyzed within the scope of this study. By isolating the hazard from the runout of existing landslides, we were able to consider if and when a threat was predicted for each building, and if this threat was realized. To evaluate model performance, two different validation criteria were created to identify the significance of i) threats predicted in any previous epoch, and ii) threats predicted in epoch (n-1). These criteria, which become increasingly restrictive, can be represented as:

(i) where R > 0 in any previous epoch, and where P = 0 in epoch (n-1), and
(ii) where R > 0 and P = 0 in epoch (n-1).

where R is the building’s modelled threat score, P is a Boolean variable indicating the presence or absence of a building within a mapped landslide, and n is the mapping and modelling epoch.

## Results

### Geographical patterns in hazard and exposure

#### Regional patterns of threat from landslides

The majority (65%, *n* = 129,201) of mapped landslides across all 14 mapping epochs from 2014–2020 are concentrated within the Middle Mountain region (MMR) (42%) and High Mountain region (HMR) (23%), with further localized concentrations in both the Hill and Siwalik regions (Figs 1a and 2a). These regional patterns in landslide concentration are largely consistent across all mapping epochs. In line with other comparable studies examining post-seismic landslide activity [19, 51, 52], the landslide inventories contain both coseismic landslides that have continued to evolve and runout downslope after the earthquake [1, 40, 52], and new rainfall-triggered landslides [15, 19]. Landslides are widely distributed across the landscape, but coseismic landslides broadly occur high on valley walls or at ridge crests, commensurate with typical locations of coseismic slope failure [38, 50], and landslide debris commonly extends downslope into the drainage network via post-earthquake remobilization [40, 58].

**Fig 3:**
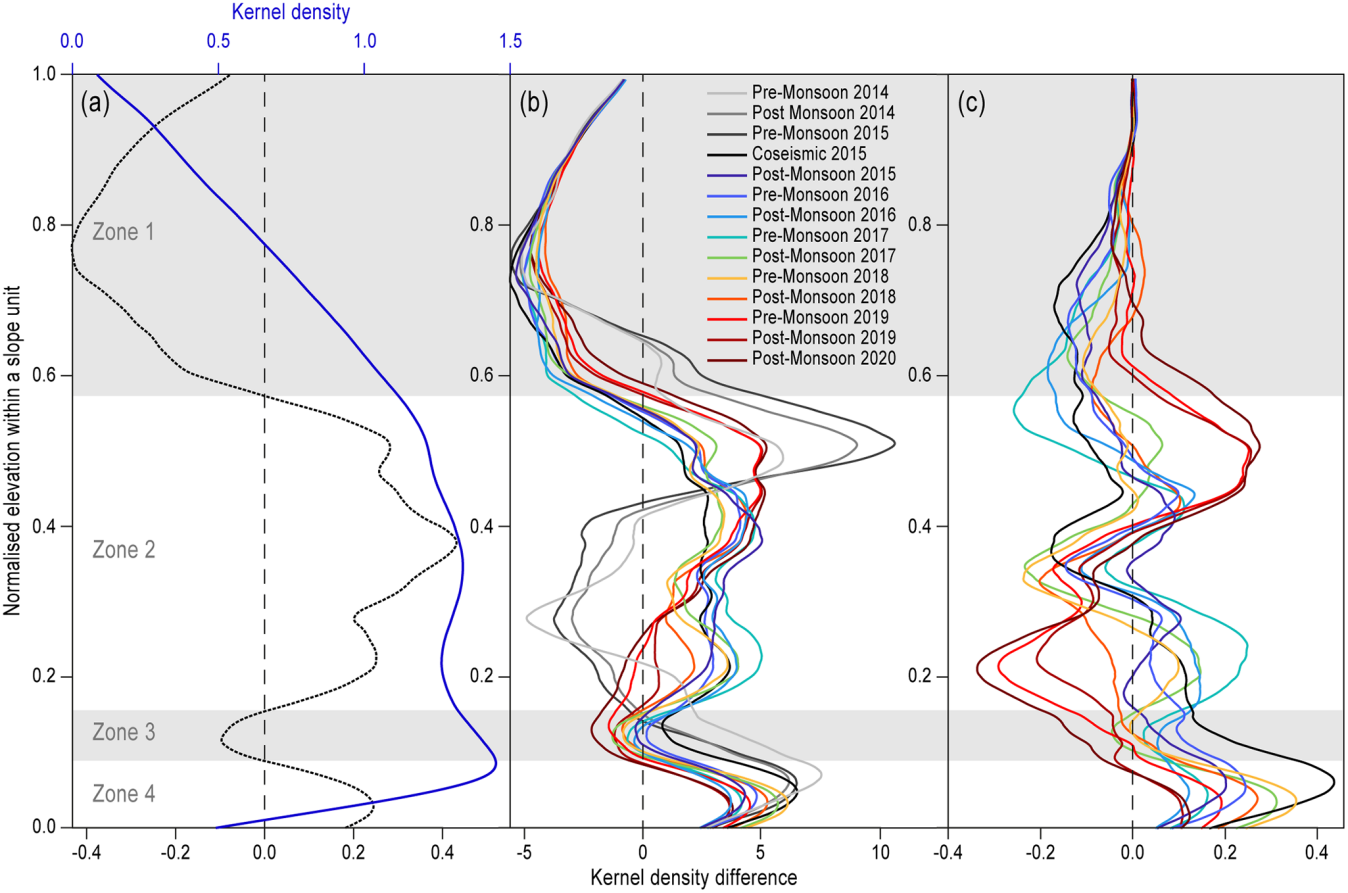
Trends in the relative locations and distributions of under-threat buildings as a function of normalized elevation within slope units (*Z’*_*SU*_). **a**) Profiles of the distribution of all buildings in the 14 earthquake-affected districts with normalized elevation within slope units (blue line, upper x axis), and distribution of under-threat buildings relative to the distribution of all buildings (grey line, lower x axis). The latter values show the difference in kernel density estimation between the full building distribution and that of under-threat buildings only. Buildings are present across the full range of elevations, but most buildings are concentrated in mid- to lower-slope positions between normalized elevations of 0.1 to 0.6. By contrast, under-threat buildings are concentrated in mid-slope positions (normalized elevations of 0.15–0.6) and hillslope toes or valley floors (normalized elevations of less than 0.1). **b)** Profiles of the distributions of under-threat buildings per epoch relative to the whole building catalogue. Threat is determined by intersection of building locations with the potential runout areas for each of the landslide mapping epochs shown. **c)** Profiles of the distributions of under-threat buildings, as in panel (b), but relative to the post-seismic under-threat building population.

Buildings, by contrast, are concentrated within the Hill region (81% of all buildings assessed), predominantly within the Kathmandu Valley conurbation, with smaller peri-urban centers often adjacent to transport corridors (Fig 2b). Notably, less than 0.3% (*n* = 2,348) and 10% (*n* = 107,493) of the buildings lie within the HMR and MMR, respectively, where landsliding is focused. The distribution of buildings within slope units, defined by their *Zˊ*_*SU*_ (Fig 3a), shows a general trend of increasing numbers of buildings towards mid- to lower-slope normalized elevations, with a decrease in the number of buildings in the lowest slope positions.

Over the 14 epochs that cover our 2014–2020 study period, approximately 2% of all buildings (23,330 unique buildings and 117,849 individual building instances) were found to be under threat from the runout of landslide material in at least one epoch (Fig 2c and 2d), and about 0.2% (2,131 buildings) were directly impacted by landslides within our inventories. This small proportion of the total stock of 1.1 million buildings still represents a significant population under threat from post-earthquake landsliding, and one that importantly was identifiable once the coseismic landslide footprint was defined [59]. Considering the whole study area, coseismic landslides affected 0.62% of the land area [1], and the equivalent area of modelled potential runout from those landslides was 2.5% [60]. Because c. 2% of buildings were modelled as being under threat, this spatial coincidence of buildings and landslide runout occurred marginally less frequently than if both were randomly distributed. Buildings are therefore in general, and unsurprisingly, built in relatively but not totally safe locations.

More than half of the buildings modelled to be under threat (*n* = 13,634) reside within the MMR (where 27% of all slope units had buildings under threat from landslide runout, compared to 2.4% within the HMR). Under-threat buildings within the MMR were more widely distributed across hillslopes (sample standard deviation of normalized elevation *S* = 0.19, and sample mean $\bar{\text{x}}=0.34$) as compared to within the HMR (*S* = 0.09,$\bar{\text{x}}=0.11$). Although the MMR had a higher number of buildings under threat from runout than the HMR, the proportion of buildings under threat were roughly similar (13% and 15%, respectively). Within the HMR, overall levels of modelled threat were high and pervasive, so where houses were present levels of threat were also high (21% of under-threat buildings at Moderate or High Threat). Whilst the MMR contains 43% of all landslides and 58% of all individual under-threat buildings, a higher proportion of buildings are located away from areas with landslide hazard.

Model performance against the two validation criteria showed variability both between epochs and between criteria. We observed a large discrepancy in prediction accuracy immediately following the earthquake, where criterion (i) can be met multiple times for the same building in the subsequent epochs.

For criterion (i) the model correctly predicted the realization of threat 1010 times for 922 buildings (mean of 67% prediction accuracy across all epochs, maximum per-epoch prediction accuracy of 78%); for criterion ii, the model correctly predicted threats 897 times for 803 buildings (mean of 60% prediction accuracy across all epochs, maximum per-epoch prediction accuracy of 74%). For each criterion and epoch, the model predicted landslide threat over a range of landscape locations, normalized elevations and contributing upslope areas. The higher scores for criterion (i) show that threat can return to buildings, which may reflect a legacy effect related to periods of dormancy and reactivation, or may simply represent sensitivities in our mapping and runout modelling.

These data show that: 1, the model can reasonably accurately predict the locations of buildings under threat from runout from landslides; 2, threat can take time to be realized, and unrealized threat may reflect either poor model performance or simply the time required to mobilize and transport sediment downslope (for example, we note that only 6% of buildings with a predicted threat score immediately following the earthquake have had that threat realized within the following five years); 3, our modelling could have provided *a priori* warning for over 900 houses subsequently impacted by landslides; and 4, presently unrealized modelled threat will in part reflect lagged impacts of the Gorkha earthquake and subsequent changes to landslide dynamics. Realized threat was more prevalent within locations with higher proximity to channels and within lower slope positions.

Examination of unpredicted threat, where runout from mapped landslides impacted buildings and was not simulated within the modelled runout from previous epochs, revealed a number of key causes or conditions for failed prediction. Landslide retrogression and rapid expansion of the margins of mapped landslides averaged across all epochs accounted for >80% of all failed predictions. The Flow-R model also underestimated flow extents in low gradient valley floors and areas of rapid gradient change, for example where steep gullies joined or transitioned into larger shallow gradients channels. In these locations the model failed to simulate mapped landslide extents, especially some of the large lobe deposits that tended to occur in these areas.

#### Hillslope-scale changes in patterns of threat from landslides

Analysis of the landscape position of buildings threatened by landslide runout revealed that the threat generally increases with distance downslope. This hillslope-scale pattern can be subdivided into four zones (Fig 3a). Runout from landsliding in upper slope positions (Zone 1) is generally directed away from traditional ridge-top settlements, which predominantly results in low threat to buildings in these areas. More complex patterns of threat from landslides are observed in mid-slope positions (Zone 2), where dispersed landsliding can coincide with dispersed valley-wall settlement patterns. Here threat from landslides is contingent on precise landslide location and runout pathway, especially if those pathways are across open and occupied hillslopes, outside of the drainage network. In Zone 3, lower slope positions exhibit relatively low levels of threat from landslides compared to the overall landscape. Here steeper gorge wall topography tends to channelize runout into the largely uninhabited drainage network. Below in Zone 4, sediment fan deposits, often situated at the base of drainage channels, are commonly the only available land for development, and so experience a frequent coincidence of modelled landslide runout and buildings, generating isolated but concentrated areas of comparatively high threat from landslides.

#### Hillslope-scale evolution of threat from landslides through time

Comparing the normalized elevation of under-threat buildings by slope unit, we see clear pre-seismic, immediate post-seismic (2015) and later (post-2017) patterns (Fig 3b and 3c). Threat from pre-seismic landslides is more localised within the landscape than from post-seismic landslides, with minimal threat in mid-lower slope positions. There are notable differences in Zone 2 between pre-, co-, and post-seismic under-threat buildings, where patterns of threat appear to have shifted to different parts of the slope. Coseismic landsliding also led to a net increase of under-threat buildings, particularly in lower slope positions, while from 2017 onwards, threat has tended to focus in Zone 4 in the lower 10% of slope positions (Fig 3c). Relatively higher or lower concentrations of under-threat buildings occupy largely consistent positions within the landscape through time (Fig 3a and 3b), but around these areas of evolving threat can be identified (Fig 3c). These include a progressive downslope shift in threat driven by runout from landslides above. Presented in terms of normalised elevation within slope units, over the period 2015–2020 there was a downward shift in the peak in threat within the per epoch kernel density plots of 0.05 in normalised elevation, which corresponds to a decrease in elevation of about 230 m, or equivalently a translation down slope of about 320 m for a hillslope in the HMR. Post-seismic movements of threat within the first two years following the earthquake were found to be one and half times greater than those observed over the full study period; although the first two post-seismic years witnessed the most change, these progressive downward patterns have continued to evolve for at least five years after the earthquake. This result shows the need for both high resolution and longitudinal landslide mapping to capture the variability and trends in threat from post-seismic landslide hazard.

### Landscape-scale evolution of threat from landslides through time

**Fig 4:**
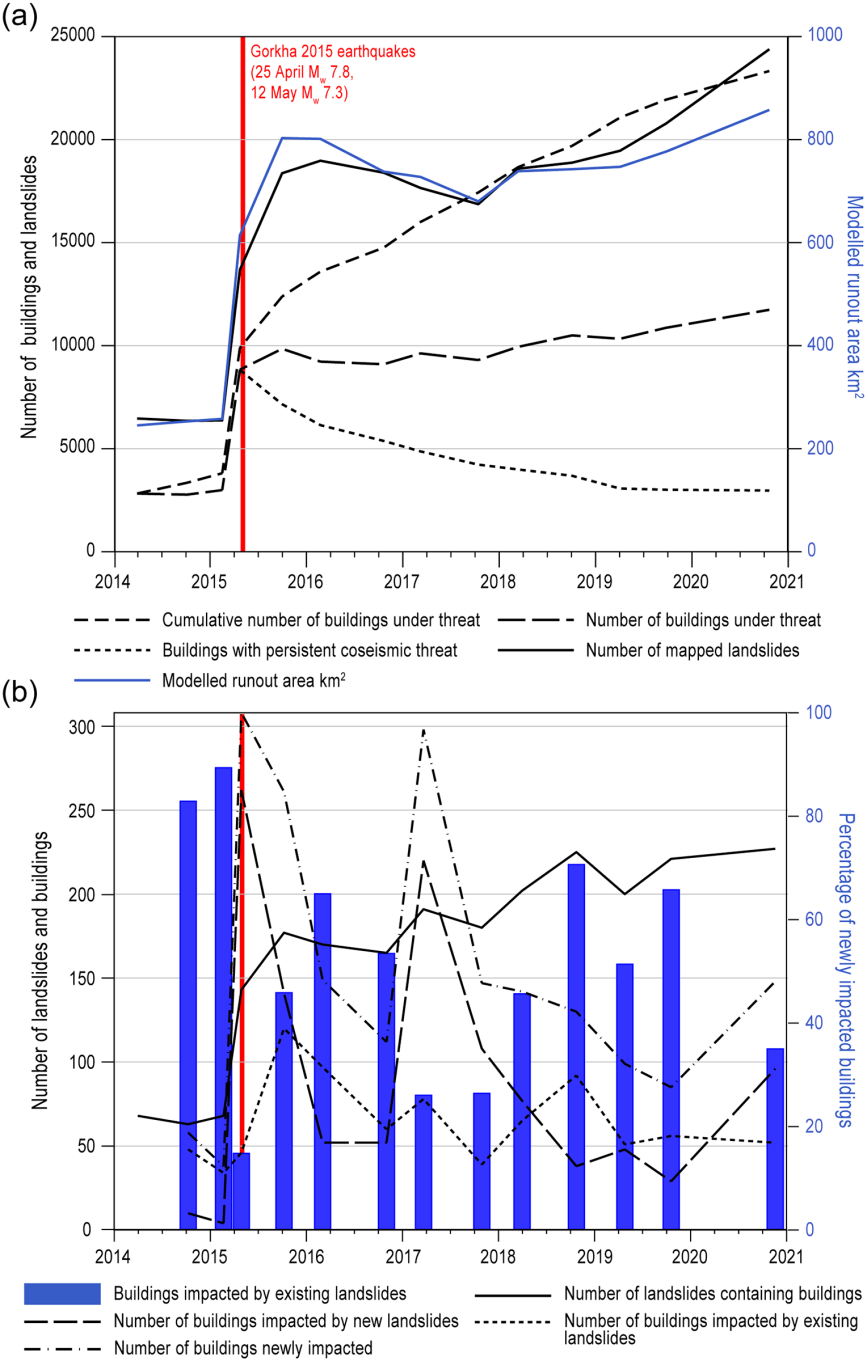
Temporal trends in mapped landslide hazard and the threat from modelled landslide runout. **a)** Number of buildings that fall within modelled runout area through time and hence are considered under threat, shown as the cumulative count of individual buildings under threat (medium-dashed line), the total per-epoch number of buildings under threat (long-dashed line) and the number of buildings persistently under threat since the earthquake (short-dashed line). In addition, the number of mapped landslides (modified from [1]) and the associated modelled runout area (modified from [40]) are displayed. **b)** The changing number of buildings impacted by newly mapped and existing landslides per epoch. Black solid line shows the per-epoch total number of landslides that were found to have impacted buildings. Blue bars show the percentage of newly-impacted buildings that were impacted by existing landslides (right-hand scale). The long-dashed line represents the number of buildings impacted by new landslides and the short-dashed line represents the number of buildings impacted by existing landslides. The dotted-dashed line represents the number of buildings newly impacted by landslides.

We observe trends through time in both the location and frequency of mapped landslides, which in turn alter where runout may later occur. By 2020, the total number, area, and runout of landslides had not returned to pre-2015 levels (Fig 4a) [1, 40]. This has resulted in a persistent threat to buildings: 34% of the c. 9000 buildings under threat from runout by coseismic (2015) landslides remained so until at least the 2020 monsoon (Fig 4a). The persistence of landsliding has driven a steady increase in the cumulative number of individual buildings under threat (Fig 4a), meaning that an assessment of risk immediately after the earthquake cannot capture all areas that will experience risk later; 55% of all buildings threatened by runout were in locations that only became under threat as a result of new post-seismic landslides or post-seismic changes to the footprint of mapped landslides. Whilst we observe a diminishing incidence of threat posed by coseismic landslides through time, this reduction is more than offset by both newly under-threat areas associated with newly-formed landslides, and in places a return of threat associated with recurrent landslides as also observed by [52].

Our modelling suggests that the per-epoch number of buildings under threat from landslide runout increased after the 2015 monsoon, was sustained and then increased from 2018 onwards, so that post-monsoon 2020 experienced the highest number of under-threat buildings of any epoch (Fig 4a). This pattern tracks the evolution of the number of mapped landslides and their modelled runout area (Fig 4a). Comparing 2014 (pre-earthquake) and 2020, we observe a 203% increase in the number of buildings under threat for the first time (1373 compared to 453), a 178% increase in the number of buildings impacted by newly mapped landslides (50 compared to 18) and a 456% increase in the number of buildings impacted by a previously mapped landslide (178 compared to 32). In total, the proportion of all buildings under threat from landslide runout increased from 0.3% in 2014 to 0.8% immediately following the earthquake and to 1.1% in 2020, equating to an additional 8,754 buildings in 2020 compared to 2014.

**Fig 5:**
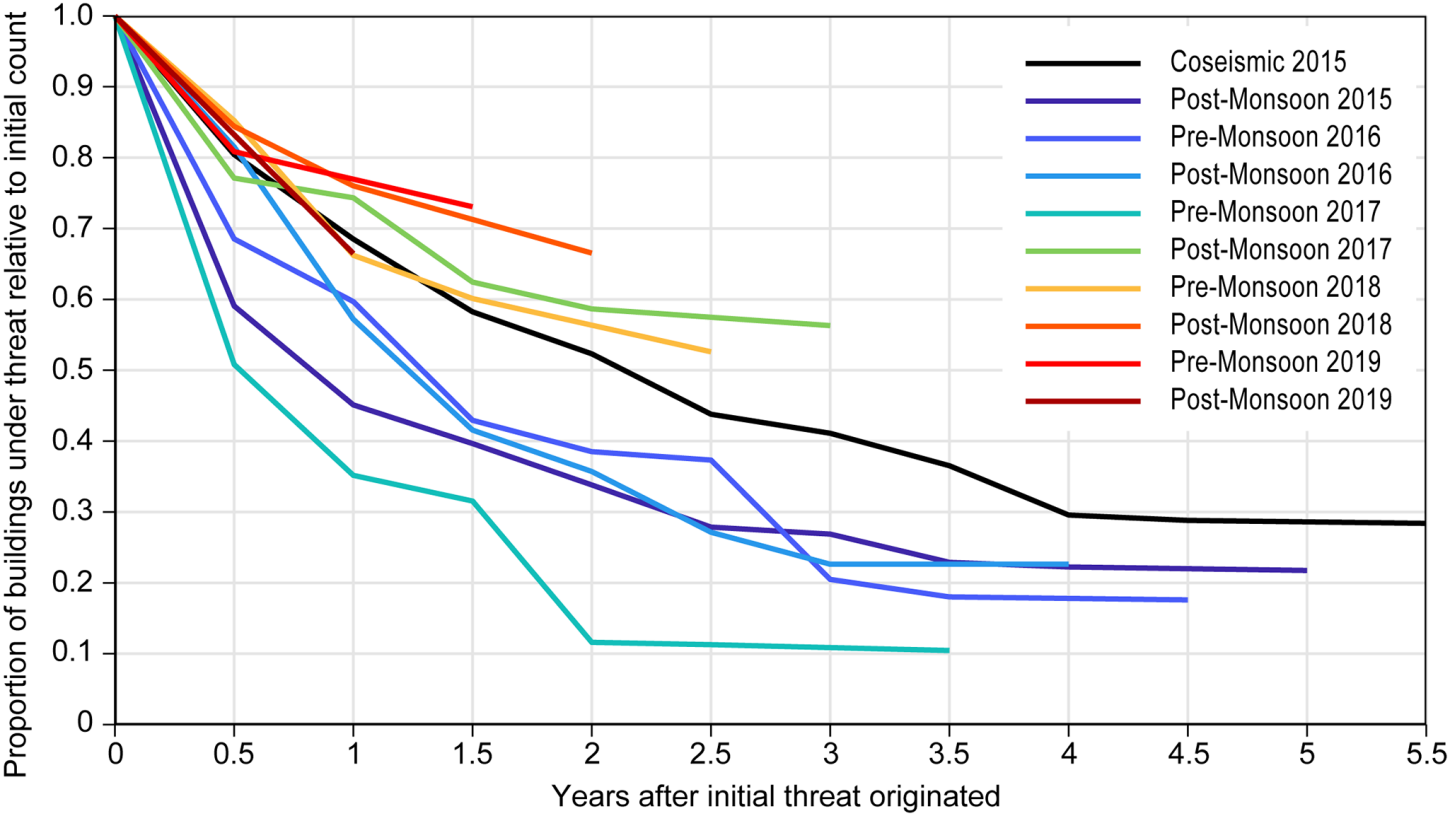
Persistence of modelled threat to buildings through time shown by epoch the threat originated. Numbers of buildings under threat are represented as the proportion of the total number in the source epoch, under threat in each subsequent epoch.

The number of buildings with threat originating in each epoch, and the persistence of this threat through time, shows that threat originating from coseismic landslides has persisted within the landscape for longer and has declined less rapidly than in the epochs that immediately follow (Pre-monsoon 2016 to Pre-monsoon 2017) (Fig 5). We find that threat originating in this period following the earthquake remained in the landscape for shorter periods of time, with more rapid reductions in the proportion of buildings under threat. From post-monsoon 2017 we note a more consistent and less rapid decline in the proportion of buildings at threat in subsequent epochs.

### Different forms of threat from post-earthquake landslide hazard

Considering the first time each building was impacted by a landslide, we can separate those instances where the source of the hazard was a newly-formed landslide from those impacted by runout from or growth in an existing landslide. Between post-monsoon 2014 and post-monsoon 2020, 838 buildings were impacted by existing landslides and 1,137 by new landslides. New landsliding was therefore the dominant source of hazard during and immediately following the Gorkha 2015 earthquake, but existing landslides still threaten significant numbers of buildings.

We observe a lag in time between the occurrence of the peak in numbers of buildings impacted by new landslides (long-dashed line Fig 4b) immediately following the earthquake in 2015 and again pre-monsoon 2017 as compared to existing landslides (short-dashed line Fig 4b) post-monsoon 2015 and post-monsoon 2018, suggesting a shift from hard-to-predict risks associated with new landslides to largely predictable risks associated with runout from existing landslides. These numbers can be dominated by single large landslide events that affect many buildings, but most incidences involve a single or small numbers of buildings. A notable exception occurred pre-monsoon 2017, which was dominated by two damaging landslides that together affected 113 buildings. We also note that new post-earthquake landslides, including these two large events, impacted a combined total of 351 buildings, comparable to the 356 impacted by coseismic landslides on the day of the 2015 earthquake, suggesting that the magnitude of threat from co- and post-seismic landslides is comparable.

### Scales over which exposure to the threat posed by landslides varies

A key motivation for combining mapped landslide distributions with a forward-looking runout model is to rapidly identify buildings that may be exposed to future landslide impact after an earthquake. Analysis of the per-epoch realization of these predictions—that is, instances where a building that fell within a modelled runout area was subsequently impacted by runout from an existing landslide—shows that the up to 78% of locations were successfully predicted *a priori* by the end of our study period. Across all the epochs analysed, for buildings where threat was both modelled and realised, 55% were impacted within one year of mapping, 72% within two years, and 84% within three years. This lag in the realization of modelled threat provides a valuable window of opportunity to direct mitigation efforts and provides a first-order estimate for timescales over which debris runout is likely to impede reconstruction over large areas [61].

**Fig 6:**
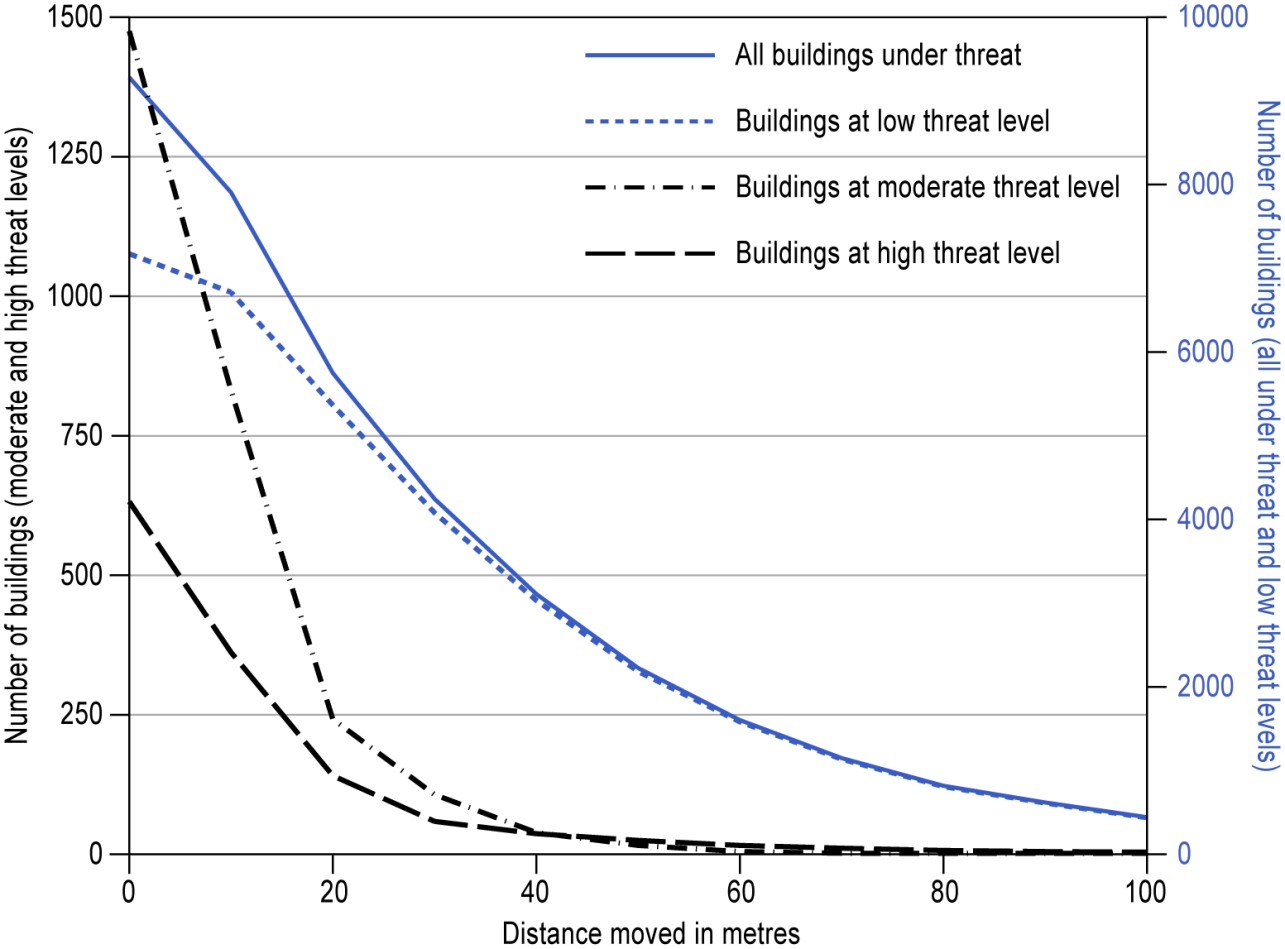
Reductions in the number of buildings (total and per threat classification) with a Flow-R runout susceptibility value within increasing buffer distance around each building. Buffers were created around each building and the minimum runout susceptibility value within each buffer was used to assess whether the building was still under threat from debris runout.

The granularity of the modelled runout susceptibility (which is done on a 10 x 10 m grid) and building locations also enables us to assess the spatial scales over which exposure varies, and thus the potential reduction of threat by small-scale changes in building location. Changes in location of as little as a few tens of meters can substantially decrease the number of buildings under threat (Fig 6). This effect is especially pronounced for buildings at high (runout susceptibility ≥0.5) and moderate (runout susceptibility of 0.1 to 0.5) threat levels. For example, moving buildings by as little as 20 m could reduce the number of properties under high threat from landslide runout by 78%, and those under moderate threat by 84% (Fig 6). This reflects the strong role of topography in confining runout, sediment remobilization and deposition into elongated but narrow areas. These results are complicated, of course, by local constraints on relocation as a result of competing risks, land tenure, and land suitable for construction, as well as uncertainties in modelled susceptibility values. Nevertheless, simple principles to guide local risk sensitive land-use planning and building location (e.g., [5]) may thus hold considerable benefit for reducing risk whilst limiting the social, political and economic complexities of wholesale settlement relocation [23].

## Discussion: Implications for post-earthquake disaster risk reduction

Across the region impacted by the Gorkha earthquake, we show a highly variable exposure to the threat posed by landslides. This variability is likely to also occur after future mountain region earthquakes, and so the patterns identified hold value in anticipating the impacts of future events. Our analysis shows that high-resolution landslide runout and building location data are essential in fully understanding exposure and risk [39, 40]. The majority of landsliding (65% of all mapped landslides) occurred in the MMR and HMR of Nepal, which contains only 10% of the total buildings in the 14 earthquake-affected districts. Furthermore, the dispersed nature of rural housing has resulted in limited numbers of under-threat buildings, with only ~2% of buildings in our catalogue modelled as being under threat over the period 2014–2020. The intersection between the distributions of landsliding and buildings produces a non-random pattern of threat with both regional- and local-scale trends. These intersections occur less frequently than if they were randomly distributed, so that buildings are generally but not always located within safe positions within the landscape.

At the scale of individual hillslopes, we identify clear patterns in exposure to the threat from landslides, and a progressive spatial shift in that threat through time. There has been a distinct increase in the proportion of buildings under threat between ridge-top positions and locations further down slope, which we attribute to both landslide runout threatening those downslope areas and the preferential location of buildings at certain slope positions. The within-slope locations of buildings under threat show a shift in their relative position between pre- and co-seismic periods, suggesting that locations that had previously been relatively safe were no longer so. This could pose challenges where communities’ experience and knowledge of relatively safe versus hazardous locations may predominantly reflect the threat of rainfall-triggered landslides which have a different distribution. Our results also highlight a gradual shift of landslide threat to lower landscape positions over the study period (Fig 3c). Prior work [1, 40] has revealed a pattern of downslope runout channelisation, and therefore the topographic containment of hazard away from buildings with distance downslope. Ultimately, where such channels run into the valley bottom, localized threat can be amplified as these areas are often the only inhabitable land, meaning that buildings there experience some of the highest levels of threat. Numerous examples of destructive long-runout channelized debris flows from failures higher up on valley walls have been documented in the earthquake-affected districts since 2015 (e.g., [4]), mirroring other studies of evolving landslide hazard following large earthquakes [3, 7, 62, 63].

Our research highlights that, although buildings and coseismic landslides may not be initially coincident, the evolution of landsliding, predominantly during the monsoon months, generates threat where runout tends to focus hazard on preferentially inhabited parts of the landscape. This finding addresses the priorities set out by [61], who stressed the need to clarify up- and down-stream linkages where hazards and impacts appear widely separated. Importantly, the threat from landslides on the day of the earthquake does not include all locations that may experience threat in the future, and so initial landslide maps—even if they are substantially complete- will tend to underestimate the total number of buildings under threat. Post-earthquake threat from landslides results from a combination of runout from existing landslides and both footprints and runout from newly-occurring landslides. We observe diminishing threat from coseismic landslides over time but find that this reduction has been more than offset by the threat from new landslides (Fig 4a). Therefore, the total caseload of under-threat buildings has increased with time after the earthquake. Policies that put time limits on the provision of support for reconstruction for damage ‘as a result of the earthquake’, specifically new building construction and relocation sites, therefore need to consider this multi-annual evolution of post-earthquake hazard, which in the case of the Gorkha earthquake has persisted for at least 5 years. It is important to note, however, that full assessment of the post-earthquake evolution of threat would require comprehensive data on new construction and relocation sites, which are not currently available.

The lag in the realization of modelled runout from landslides provides a window of opportunity to mitigate landslide risk that ranges from periods of months to years. The position of buildings that became under threat after the earthquake has evolved continuously (Fig 3c) over multiple years [64–66]. Where the modelled threat was realized, this occurred most often before the next mapping epoch (<6 months), suggesting that once destabilised, landslide debris tends to run out within a short period of time. An ability to rapidly identify and support areas experiencing landsliding is therefore critical in reducing post-earthquake landslide risk.

The threat to buildings from cosesimic landsliding is more persistent than the threat from landsliding originating in the two years following the earthquake; after three years, the number of buildings under threat from coseismic landslide runout was halved, whereas for immediate post-sesimic landslides, the same reduction has been achieved over two years. Threat associated with runout from coseismic landsliding continued to be realized more than five years after the event, with almost all other epochs having the number of buildings with threat realized tending to zero within two years. The differing trajectories of threat originating from coseismic and post-seismic landsliding suggest differences in the stability of those landslides, their positions in the landscape, or their propensity to remobilize, and future research should seek to address these issues. Whilst here we focus on dynamic landslide hazard, it is clear that capturing dynamic exposure, including the effects of reconstruction and relocation, is equally important but is a critical gap in the current management of post-earthquake risk.

## Conclusions

The threat from landsliding to buildings following the 2015 M_w_ 7.8 Gorkha earthquake has been shown to remain for at least five years, as a composite of persistent threat from coseismic landslides and elevated levels of new landsliding following the event. Locations within the landscape under threat were found to be highly dynamic, driven by systematic patterns in new landsliding and the evolution of coseismic and post-seismic landslides, with increasing levels of threat concentrated within the bottom 10% of slopes, coincident with the location of many buildings. Modelling the susceptibility of locations to landslide runout from mapped landslides provided a forward-looking perspective on the landscape and building locations under threat in future, providing a valuable window for planning and mitigation measures. This approach enabled differentiation between easier to predict threats from existing landslides and harder to predict threats from new landslides, where we identified *a priori* the locations of 78% of buildings that were impacted by landslide debris.

Exposure to the threat from landslides varies from significant to negligible over short distances and so a significant portion of threat can be reduced via short distance relocation (c. a few 10s of m), rather than wholesale settlement relocation. This highlights the importance of high-resolution data on both the changing hazard and exposure to support reconstruction decisions, to assess the need for relocation, and to identify suitable alternatives.

Post-earthquake reconstruction must be supported by a rolling assessment of risk. Our results suggest that attempts to quantify broad timescales for post-seismic ‘recovery’ and an associated reduction of risk should only be made with caution, notably in regions with a comparable variability in type, frequency and magnitude of landsliding. Assessing this change in risk requires high-resolution landslide runout and building location data to fully assess risk.
